# Progesterone Enhances Sensitivity of Ovarian Cancer Cells to SN38 Through Inhibition of Topoisomerase I and Inducing Ferroptosis

**DOI:** 10.1002/cnr2.70202

**Published:** 2025-04-24

**Authors:** Takahiro Koyanagi, Yasushi Saga, Yoshifumi Takahashi, Kohei Tamura, Eri Suizu, Suzuyo Takahashi, Akiyo Taneichi, Yuji Takei, Hiroaki Mizukami, Hiroyuki Fujiwara

**Affiliations:** ^1^ Department of Obstetrics and Gynecology, School of Medicine Jichi Medical University Shimotsuke City Tochigi Japan; ^2^ Division of Genetic Therapeutics Center for Molecular Medicine, Jichi Medical University Shimotsuke City Tochigi Japan

**Keywords:** ferroptosis, irinotecan, membrane progesterone receptor, non‐genomic action, ovarian cancer

## Abstract

**Background:**

Progesterone rapidly induces ovarian cancer cell death through non‐genomic actions mediated by the membrane progesterone receptor (mPR).

**Aims:**

We investigated the combined effects of progesterone and SN38, an active metabolite of irinotecan, on ovarian cancer cells.

**Methods and Results:**

mPR‐positive and PR‐negative ovarian cancer cell lines were utilized in experiments. Tumor cells were exposed to SN38 or cisplatin for 48 h following exposure to progesterone for 30 min. The viable cell counts were measured using a colorimetric assay and the expression of topoisomerase I (TOPO‐I), the direct target of SN38, was observed with or without exposure to progesterone. Moreover, we investigated the relationship between several types of programmed cell death and the SN38 sensitivity enhancement effect of progesterone using specific cell death inhibitors. The chemosensitivity to SN38 was 8.7‐ to 26.0‐fold higher with the administration of progesterone than that without (*p* < 0.01), but not to cisplatin in ovarian cancer cells. Progesterone suppressed the expression of TOPO‐I mRNA by less than 50% (*p* < 0.01). Furthermore, among various programmed cell death inhibitors, only the ferroptosis inhibitor attenuated the progesterone‐induced SN38 chemosensitivity enhancement effect.

**Conclusions:**

Progesterone increased sensitivity to SN38 by suppressing TOPO‐I expression and inducing ferroptosis. The combination of progesterone and irinotecan could be a novel treatment modality for ovarian cancer.

## 1 Introduction

In the United States, the sixth leading cause of death from cancer in women is ovarian cancer. It is estimated that approximately 13 000 women will die of the disease [1]. Over 50% of the patients are diagnosed at an advanced stage with more peritoneal dissemination and much ascites owing to the absence of symptoms at an early stage. The standard treatments for advanced stage ovarian cancer are primary debulking surgery and intensive adjuvant chemotherapy with platinum, taxane, and bevacizumab followed by maintenance therapy including bevacizumab and poly adenosine diphosphate‐ribose polymerase (PARP) inhibitor [2, 3, 4]. Because ovarian cancer is relatively chemotherapy‐sensitive, these intensive therapeutic interventions result in complete remission in approximately 80% of the patients. However, the antitumor effects are often transient, and over 50% of the patients exert pelvic recurrence with less chemosensitivity, ultimately leading to death due to disease progression [2, 3, 4]. Although novel molecular targeted agents show antitumor effects to some extent, specific adverse effects and economic burdens remain significant challenges that need to be addressed. Therefore, current treatments have limitations and new treatments, particularly those that are effective against platinum‐resistant recurrent cases, are urgently needed.

Irinotecan is an anticancer agent that acts on topoisomerase I (TOPO‐ I) [5]. Irinotecan is metabolized in the liver and converted to the active metabolite SN38, which exerts the antitumor effect. It is one of the agents that is effective against platinum‐resistant and taxane‐resistant recurrent ovarian cancer and is usually used for second‐line or later chemotherapy for platinum‐resistant recurrent ovarian cancer [6]. Resistance to platinum‐derived drugs is mainly due to an increased antioxidant defense of the ovarian cancer cells owing to an increased expression/activity of NRF2/KEAP1 signaling, the key factor in redox maintenance [7, 8]. It is also reported that SN38 can inhibit this signaling, restoring platinum response in resistant cancer cells [9].

Progesterone is a steroid hormone mainly synthesized in the corpus luteum of the ovary. Progesterone forms a complex with intranuclear progesterone receptor (PR) proteins. The complex binds to a DNA promoter site in the nucleus and regulates the expression of several genes [10]. In oncology, progesterone preparations, such as medroxyprogesterone acetate, are used clinically as an inexpensive treatment option with fewer side effects for breast and endometrial cancers [11, 12]. The membrane progesterone receptor (mPR) family was identified as a PR specifically expressed on the cytoplasmic membrane in 2003 [13]. These mPRs show a rapid non‐genomic action, while the well‐known genomic action occurs more slowly through the nuclear PRs [14].

We previously reported that progesterone rapidly induces ovarian cancer cell death through non‐genomic actions [15]. In this study, we focused on combining progesterone with existing anticancer drugs, cisplatin (CDDP) and SN38, using ovarian cancer cell lines to obtain clues for a novel therapeutic strategy. Moreover, to partly elucidate the mechanism of the combined effect, we observed the conditions of apoptosis, pyroptosis, ferroptosis, and necroptosis, representing programmed cell death, which has garnered attention recently.

## 2 Methods

### 2.1 Cell Lines and Cell Culture

In the present study, specific ovarian cancer cell lines were used and a normal ovarian surface epithelial cell line was not utilized because we aimed to investigate the impact of progesterone on chemosensitivity in ovarian cancer cells rather than in normal cells. The human ovarian serous carcinoma cell line, TU‐OS‐4, was provided by the establisher [16]. The human ovarian serous carcinoma cell line OVKATE was purchased from the Japanese Collection of Research Bioresources (JCRB; Osaka, Japan). In our previous studies, these cells did not express PR but expressed various mPRs [15]. Furthermore, they showed obvious sensitivity to progesterone [15]. Both cell lines were incubated in Dulbecco's Modified Eagle Medium/F12 (Thermo Fisher Scientific Inc., Waltham, MA, USA) supplemented with 10% heat‐inactivated fetal bovine serum (Sigma‐Aldrich; Merck KGaA, Darmstadt, Germany) and 1% streptomycin/penicillin (Thermo Fisher Scientific Inc.) at 37°C in the humidified atmosphere under 5% CO_2_.

### 2.2 Colorimetric Assay

Tumor cells (1000 cells/100 μL/well) seeded onto a 96‐well plate were exposed to progesterone (160–24511; FUJIFILM Wako Pure Chemical Co., Osaka, Japan) at concentrations of 0 or 100 μM for 30 min. After removing the medium, tumor cells were exposed to CDDP (4291401A1119; Nichi‐Iko Pharmaceutical Co. Ltd., Tokyo, Japan) at concentrations of 1–32 μM for 48 h or exposed to SN38 (E0748; Tokyo Chemical Industry Co. Ltd., Tokyo, Japan.) at concentrations of 0.1–32 μM for 48 h. The viable cell counts were measured by the colorimetric assay using the Premix WST‐1 Cell Proliferation Assay System (MK400; Takara Bio Inc., Tokyo, Japan), which is considered to be more sensitive than the MTT assay and XTT assay. The absorbance was measured at a wavelength of 450 nm using a microplate reader [15]. Each value was determined as a percentage ratio to the counts of the control untreated with CDDP or SN38.

### 2.3 Quantitative Reverse Transcription‐Polymerase Chain Reaction (RT‐qPCR)

Tumor cells (5 × 10^5^ cells/2 mL/well) seeded onto a 6‐well plate were exposed to progesterone at a concentration of 400 μM for 30 min. Intracellular mRNA was extracted using RNeasy Mini Kit (74134; Qiagen, Valencia, CA, USA) following the manufacturer's instructions. The concentration of extracted RNA was determined using the Nanodrop 2000c spectrophotometer (Thermo Scientific, Wilmington, DE). RT‐qPCR was conducted using Thermal Cycler Dice Real Time System II (Takara Bio Inc.) according to the manufacturer's instructions. The PCR was carried out using 40 cycles of heating at 95°C for 15 s, 58°C for 15 s, and 72°C for 20 s. The intracellular mRNA levels of TOPO‐I were determined relative to the fluorescence signal of the glyceraldehyde‐3‐phosphate dehydrogenase (*GAPDH*). The primer sequences are as follows: GAPDH: forward: 5′‐ACCACAGTCCATGCCATCAC‐3′, reverse: 5′‐CATCACGCCACAGTTTCCCG‐3′; TOPO‐I: forward: 5′‐GAACAAGCAGCCCGAGGATGAT‐3′, reverse: 5′‐TGCTGTAGCGTGATGGAGGCAT‐3′ [15].

### 2.4 Programmed Cell Death Detection Method

Tumor cells (1000 cells/100 μL/well) seeded onto a 96‐well plate were exposed to the pan‐caspase inhibitor Z‐VAD‐FMK (ab120382; Abcam, Cambridge, UK), caspase‐1 specific inhibitor Z‐YVAD‐FMK (S8507; Selleck Biotech, Tokyo, Japan), ferroptosis inhibitor Ferrostatin‐1 (1772; Cayman Chemical, Ann Arbor, MI, USA), or necroptosis inhibitor Necrostatin‐1 (ab141053; Abcam) at a concentration of 100 μM. After 1 h, the medium was removed and then tumor cells were exposed to progesterone at a concentration of 100 μM for 30 min. After removing the medium, tumor cells were exposed to SN38 at a concentration of 1.0 μM (TU‐OS‐4 cells) or 0.5 μM (OVKATE cells) for 48 h. Viable cell count was measured using the Premix WST1 Cell Proliferation Assay System and presented as a percentage ratio to the counts of the control untreated with SN38 and programmed cell death inhibitors. More precisely, Z‐VAD‐FMK inhibits both apoptosis and pyroptosis, whereas Z‐YVAD‐FMK inhibits pyroptosis. Ferrostatin‐1 inhibits ferroptosis, and Necrostatin‐1 blocks necroptosis specifically [17]. The inhibitor concentration and reaction time were determined according to the respective package inserts.

### 2.5 Statistical Analysis

Statistical analyses were performed with EZR software (Jichi Medical University, Saitama Medical Center, Saitama, Japan). The Student's *t* test was utilized to compare the two groups. The value of *p* < 0.05 was considered to be statistically significant. All experiments were performed at least three times.

## 3 Results

### 3.1 Chemosensitivity

**FIGURE 1:**
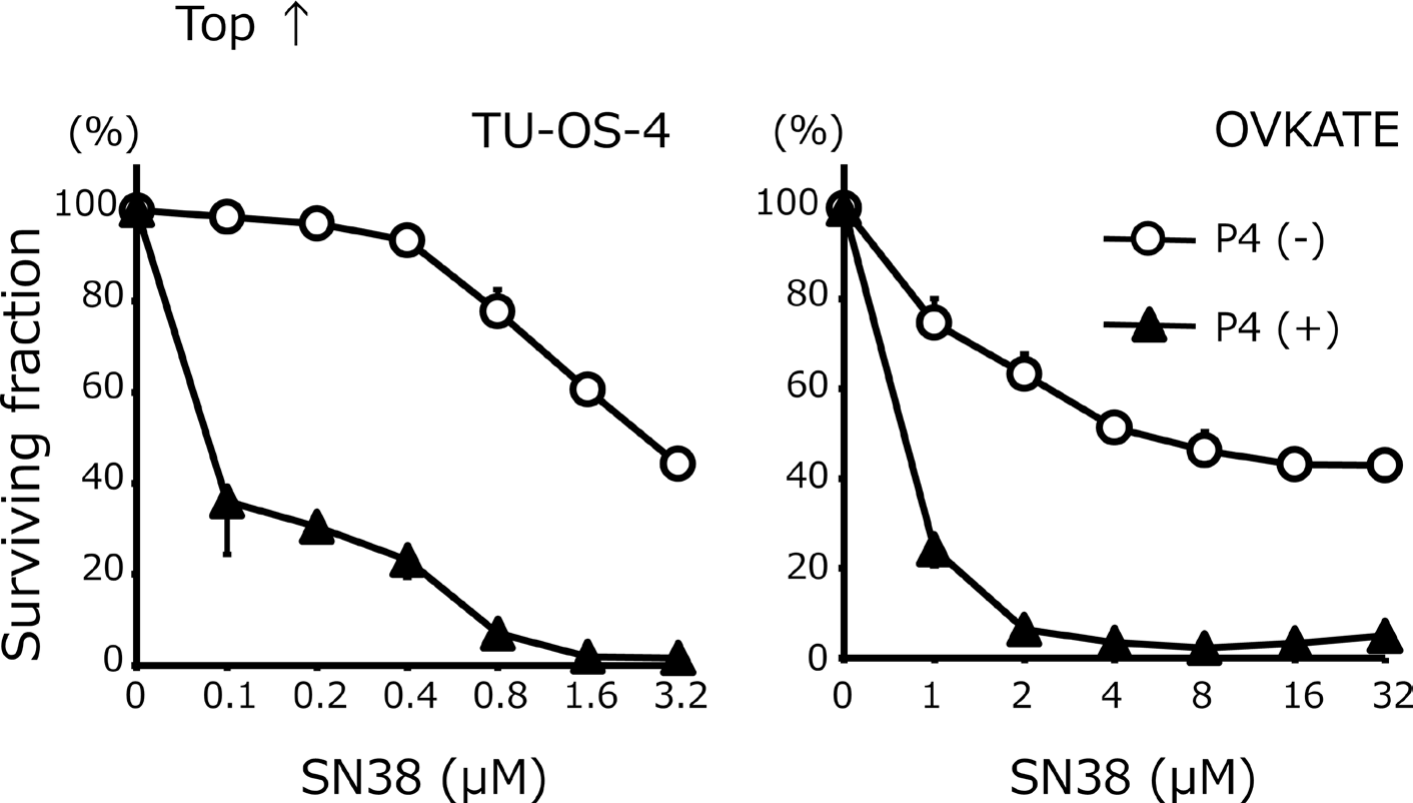
Chemosensitivity to SN38. The IC_50_ for SN38 in TU‐OS‐4 were as follows: With progesterone, 0.1 ± 0.0 μM versus without progesterone, 2.6 ± 0.1 μM (26.0‐fold higher sensitivity, *p* < 0.01). The IC_50_ for SN38 in OVKATE were as follows: With progesterone, 0.7 ± 0.0 μM versus without progesterone, 6.1 ± 2.6 μM (8.7‐fold higher sensitivity, *p* < 0.01). Data are shown as means and SD (*n* = 3). P4, progesterone.

**FIGURE 2:**
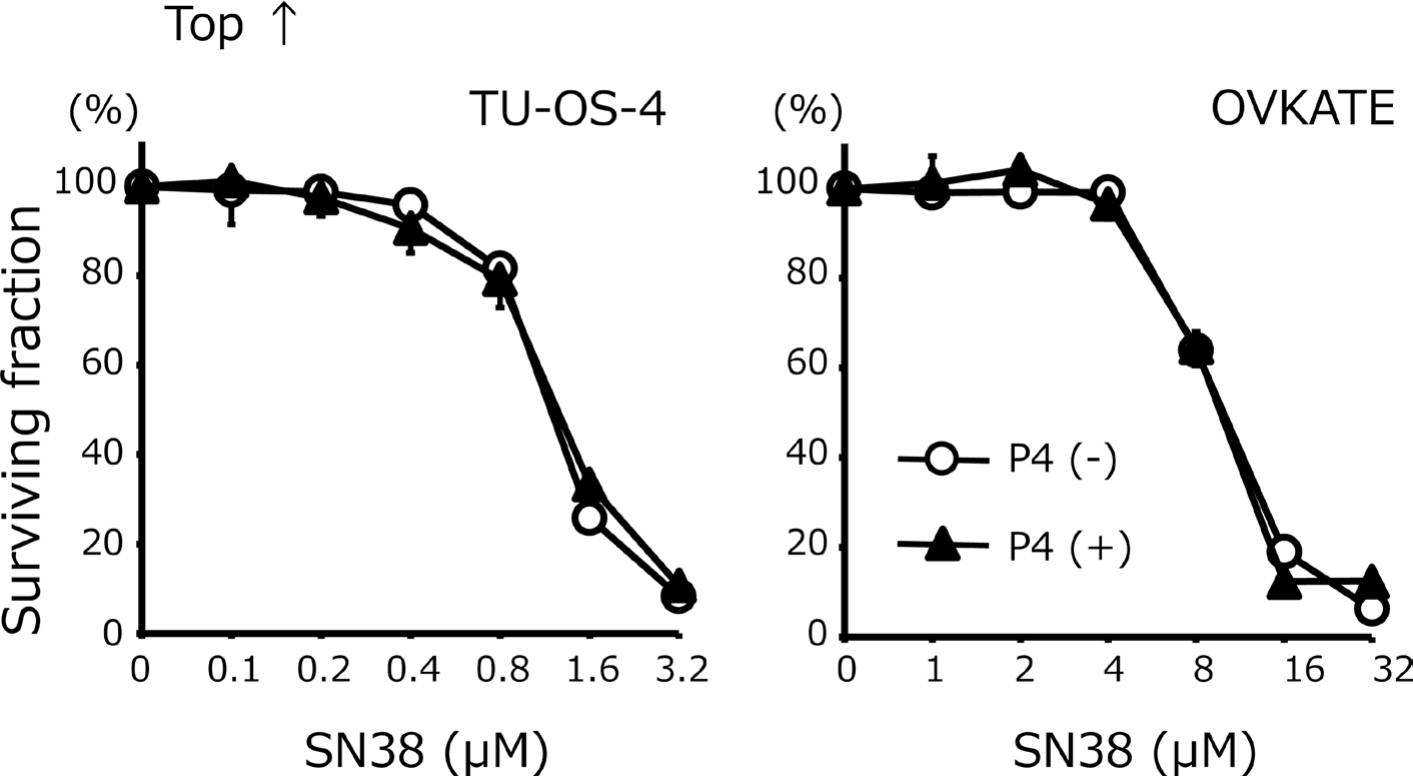
Chemosensitivity to CDDP. The IC_50_ for CDDP in TU‐OS‐4 were as follows: With progesterone, 13.5 ± 0.5 μM versus without progesterone, 12.5 ± 0.2 μM (not significant). The IC_50_ for CDDP in OVKATE were as follows: With progesterone, 10.2 ± 0.4 μM versus without progesterone, 10.5 ± 0.5 μM (not significant). Data are shown as mean and SD (*n* = 3). CDDP, cisplatin.

First, the effects of progesterone on anticancer drug sensitivity were examined. The chemosensitivity of each cell line to SN38 is shown in Figure 1. The IC_50_ for SN38 in TU‐OS‐4 cells with progesterone was 0.1 ± 0.0 μM, which was 26.0‐fold higher than that of TU‐OS‐4 cells without progesterone (2.6 ± 0.1 μM) (*p* < 0.01). Similarly, the IC_50_ for SN38 in OVKATE cells with progesterone was 0.7 ± 0.0 μM, which was 8.7‐fold higher than that of OVKATE cells without progesterone (6.1 ± 2.6 μM) (*p* < 0.01). In contrast, as shown in Figure 2, no significant difference was noted in the IC_50_ for CDDP in TU‐OS‐4 with or without progesterone (13.5 ± 0.5 μM for TU‐OS‐4 cells with progesterone and 12.5 ± 0.2 μM for TU‐OS‐4 cells without progesterone). Similarly, with or without progesterone, there were no significant differences in the IC_50_ for CDDP in OVKATE cells (10.2 ± 0.4 μM with progesterone and 10.5 ± 0.5 μM without progesterone). Collectively, these results demonstrated that progesterone increased chemosensitivity to SN38, but not to CDDP, in ovarian cancer cells.

### 3.2 TOPO‐I Expression

**FIGURE 3:**
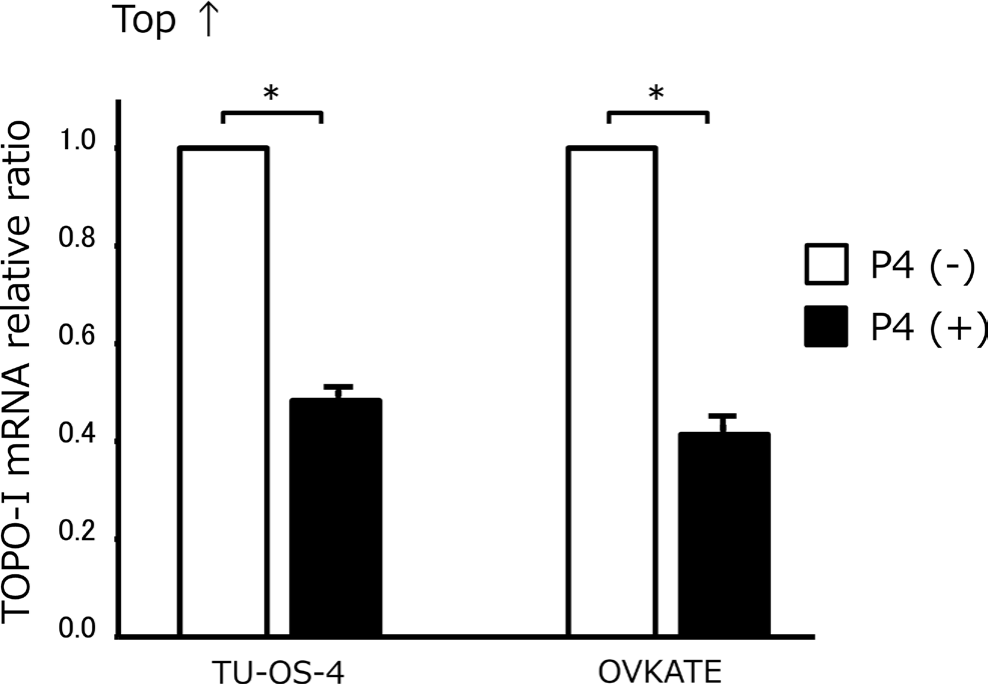
TOPO‐I expression after exposure to progesterone. The exposure to progesterone markedly decreased TOPO‐I expression in both TU‐OS‐4 and OVKATE cell lines (*p* < 0.01). Data are shown as means and SD (*n* = 3). P4, progesterone; TOPO‐I, topoisomerase I. *p < 0.01.

Next, to elucidate the mechanism by which progesterone enhances SN38 sensitivity, the expression of TOPO‐I, a direct target of SN38, was examined. The mRNA expression of TOPO‐I with or without the administration of progesterone was evaluated. Exposure to progesterone markedly decreased TOPO‐I expression in both TU‐OS‐4 and OVKATE cells (*p* < 0.01) (Figure 3).

### 3.3 Programmed Cell Death Detection

**FIGURE 4:**
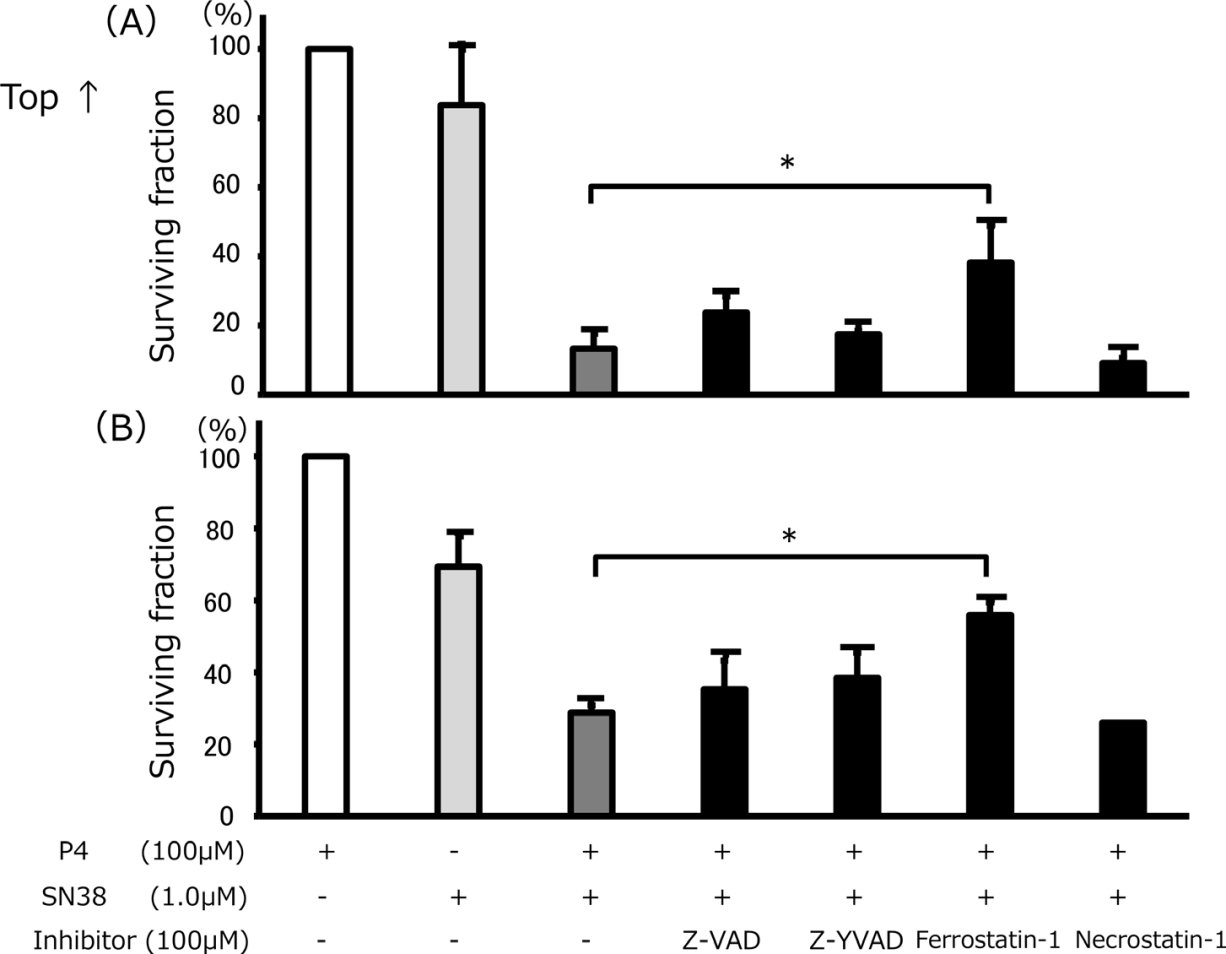
Relationship between programmed cell death and SN38 sensitivity enhancement effect of progesterone. Only ferrostatin reduced the SN38 sensitivity enhancement effect of progesterone in both TU‐OS‐4 (A) and OVKATE (B). No other programmed cell death inhibitors affected the SN38 sensitivity enhancement effect of progesterone. Data are shown as means and SD (*n* = 3). P4, progesterone. *p < 0.01.

To partly elucidate the mechanism of the combined effect, the relationship between programmed cell death and the SN38 sensitivity enhancement effect of progesterone was investigated. Notably, only ferrostatin, a specific ferroptosis inhibitor, significantly reduced the SN38 sensitivity enhancement effect of progesterone in both TU‐OS‐4 and OVKATE cell lines (*p* < 0.01) (Figure 4). However, other programmed cell death inhibitors had no effect on the augmentation of chemosensitivity to SN38.

## 4 Discussion

We focused on combining progesterone with existing anticancer drugs, especially SN38 and CDDP, using ovarian cancer cell lines to obtain clues for a novel therapeutic strategy. To elucidate the mechanism by which progesterone enhances SN38 sensitivity, we evaluated the expression of TOPO‐I, a direct target of SN38. Furthermore, to partly elucidate the mechanism of the combined effect, we investigated the relationship between programmed cell death and the SN38 sensitivity enhancement effect of progesterone.

In the present study, progesterone increased chemosensitivity to SN38, an active metabolite of irinotecan, in two ovarian cancer cell lines, whereas it did not affect the sensitivity to CDDP. Progesterone markedly suppressed the expression of TOPO‐I, a direct target of SN38. Furthermore, among various programmed cell death inhibitors, only the ferroptosis inhibitor attenuated this progesterone‐induced chemosensitivity enhancement effect. These results suggest that progesterone enhanced SN38 sensitivity by downregulating TOPO‐I expression and inducing ferroptosis in ovarian cancer cells.

We previously reported that progesterone‐induced cell death in different kinds of ovarian cancer cell lines in vitro [15]. All of these ovarian cancer cells were PR‐negative and mPR‐positive, and cell death was observed within a short period of 30 min. This rapid cellular death was therefore concluded to be a non‐genomic action of progesterone mediated by mPRs, not a genomic action mediated by PR. Because the two ovarian cancer cell lines used here were also PR‐negative and mPR‐positive, the SN38 sensitivity enhancement effect of progesterone is considered to be a non‐genomic effect.

The direct target of SN38 is TOPO‐I. TOPO‐I acts to remove abnormal twists in the double helix structure of DNA, promoting DNA replication and RNA transcription. SN38 inhibits these functions of TOPO‐I, resulting in cell death [5]. In our previous report, when the tumor suppressor gene *PTEN* was forcedly expressed in ovarian cancer cell lines, the sensitivity to SN38 was enhanced by the suppression of TOPO‐I activity and the induction of apoptosis [18]. In the current study, we measured changes in the mRNA expression of TOPO‐I with or without the administration of progesterone. Specifically, progesterone markedly suppressed the expression of TOPO‐I. Taken together, these findings indicate that progesterone suppresses the expression of TOPO‐I through a non‐genomic action mediated by mPRs to enhance the effect of SN38.

The combination therapy of CDDP and TOPO‐I inhibitors including irinotecan is widely applied in clinical practice. These synergistic effects are schedule‐dependent. Pretreatment with CDDP induces G2/M block in the cell cycle and enhances the efficacy of TOPO‐I inhibitors [19]. Ma et al. [19] also reported that there was no significant correlation between TOPO‐I activity and CDDP chemosensitivity in multiple ovarian cancer cell lines. Another report indicated that overexpression of TOPO‐I increased sensitivity to CDDP [20]. In our previous report, when *PTEN* was forcedly expressed in ovarian cancer cell lines, TOPO‐I activity was significantly suppressed, whereas the chemosensitivity to CDDP did not change [18]. In the current study, progesterone markedly suppressed the expression of TOPO‐I mRNA, whereas the chemosensitivity to CDDP did not differ with or without the administration of progesterone. The combination of progesterone and CDDP has not been shown to be effective, but the combination of CDDP, TOPO‐I inhibitor, and progesterone might be effective.

Although apoptosis is widely known as a type of programmed cell death, a variety of other kinds of programmed cell death have been reported in recent years [21]. We investigated the relationship between several types of programmed cell death and the SN38 sensitivity enhancement effect via the non‐genomic action of progesterone. Because various types of programmed cell death have similar final morphology, they are commonly differentiated using specific inhibitors for each [17]. In this study, we used specific inhibitors of apoptosis, pyroptosis, ferroptosis, and necroptosis, which are representative types of programmed cell death. Consequently, only the ferroptosis inhibitor attenuated the SN38 sensitivity enhancement effect of progesterone. These results suggest that ferroptosis is involved in the SN38 sensitivity enhancing effect of progesterone. Ferroptosis inhibitors had no effect on the cytocidal effects of progesterone or SN38 alone (data not shown), suggesting that ferroptosis is induced only by the combined use of progesterone and SN38. Our previous study has demonstrated that progesterone rapidly induces BAX expression by non‐genomic action mediated by mPRs in ovarian cancer cells [21]. BAX is a member of the Bcl‐2 family and induces not only apoptosis but also ferroptosis and pyroptosis [22, 23]. The observed effect of progesterone on augmenting SN38 sensitivity may be associated with BAX‐mediated ferroptosis.

Clinical application of PARP inhibitors, one of the molecular targeted drugs, has begun in the ovarian cancer treatment [2, 3, 4]. PARP inhibitors have been thought to block the progression of the replisome by capturing PARP on DNA, causing DNA double‐strand breaks [24]. Recently, transcription–replication conflicts (TRC) have been reported as a new mechanism of action of PARP inhibitors [25]. PARP inhibitors inhibit the function of TRC defense factors such as TIMELESS, TIPIN, and PARP1, and promote TRC, causing DNA double‐strand breaks. Notably, TOPO‐I is also one of the TRC defense factors [26]. This study indicated that progesterone suppresses the expression of TOPO‐I. Therefore, progesterone might enhance the cytocidal effects of not only irinotecan but also PARP inhibitors via TRC.

Because the prognosis for recurrent ovarian cancer is poor and irinotecan is often used for second‐line or later chemotherapy [27], augmentation of the chemosensitivity for SN38 might contribute to improved prognosis especially for recurrent cases. Another challenge is the economic burden and specific side effects of chemotherapeutic agents. Progesterone is used for a threatened premature birth and the treatment cost is relatively low [28]. Therefore, progesterone may offer a safer and less expensive treatment option for ovarian cancer. However, progesterone is rapidly disassembled in vivo by various kinds of hydroxylases synthesized in the liver [29]. Further investigations that include in vivo experiments are required to determine appropriate dosage and administration methods for progesterone.

In conclusion, progesterone increased chemosensitivity to SN38 through suppressing TOPO‐I expression and inducing ferroptosis. The combination of progesterone and irinotecan could be a novel treatment modality for ovarian cancer, ultimately leading to improved prognosis in advanced cases.

## Author Contributions

**Takahiro Koyanagi** and **Yasushi Saga:** conceptualization, funding acquisition, investigation (all experiments), writing – original draft. **Yoshifumi Takahashi**, **Kohei Tamura**, and **Eri Suizu:** investigation (programmed cell death detection). **Suzuyo Takahashi** and **Akiyo Taneichi:** methodology (programmed cell death detection). **Yuji Takei**, **Hiroaki Mizukami**, and **Hiroyuki Fujiwara:** supervision, writing – review and editing.

## Conflicts of Interest

The authors declare no conflicts of interest.

## Data Availability

The data that support findings of the research are available from the corresponding author upon the reasonable request.
